# Could Polyphenols Really Be a Good Radioprotective Strategy?

**DOI:** 10.3390/molecules26164969

**Published:** 2021-08-17

**Authors:** Shadab Faramarzi, Simona Piccolella, Lorenzo Manti, Severina Pacifico

**Affiliations:** 1 Department of Environmental, Biological and Pharmaceutical Sciences and Technologies, University of Campania “Luigi Vanvitelli”, Via Vivaldi 43, 81100 Caserta, Italy; shadab.faramarzi@unicampania.it (S.F.); simona.piccolella@unicampania.it (S.P.); 2 Department of Plant Production and Genetics, Razi University, Kermanshah 67149-67346, Iran; 3 Department of Physics E. Pancini, University of Naples “Federico II”, and Istituto Nazionale di Fisica Nucleare, (INFN), Naples Section, Monte S. Angelo, Via Cinthia, 80126 Napoli, Italy; lorenzo.manti@na.infn.it

**Keywords:** ionizing radiation, radioprotection, polyphenols, flavonoids, plant extracts

## Abstract

Currently, radiotherapy is one of the most effective strategies to treat cancer. However, deleterious toxicity against normal cells indicate for the need to selectively protect them. Reactive oxygen and nitrogen species reinforce ionizing radiation cytotoxicity, and compounds able to scavenge these species or enhance antioxidant enzymes (e.g., superoxide dismutase, catalase, and glutathione peroxidase) should be properly investigated. Antioxidant plant-derived compounds, such as phenols and polyphenols, could represent a valuable alternative to synthetic compounds to be used as radio-protective agents. In fact, their dose-dependent antioxidant/pro-oxidant efficacy could provide a high degree of protection to normal tissues, with little or no protection to tumor cells. The present review provides an update of the current scientific knowledge of polyphenols in pure forms or in plant extracts with good evidence concerning their possible radiomodulating action. Indeed, with few exceptions, to date, the fragmentary data available mostly derive from in vitro studies, which do not find comfort in preclinical and/or clinical studies. On the contrary, when preclinical studies are reported, especially regarding the bioactivity of a plant extract, its chemical composition is not taken into account, avoiding any standardization and compromising data reproducibility.

## 1. Introduction

Life on Earth has evolved in the presence of a continuous exposure to ionizing radiation (IR), whose mode of action at the biomolecular level is unique among all known mutagen and carcinogenic agents [1]. This is due to the peculiar pattern of energy deposition accompanying IR absorption at the micro- and nanometer scale [2], which is inherently nonhomogeneous, resulting in either isolated or highly clustered ionization events. As a consequence, they may generate a plethora of DNA lesions of varying severity, ranging from base damage and molecular cross-links to the most deleterious single- and double strand breaks (SSB and DSB, respectively) [3]. Indeed, cellular DNA has always been regarded as the target of choice of IR biological action because it is present in a single copy, hence any un- or mis-repaired damage can have relevant consequences, impinging on genome integrity and stability in the exposed cellular progeny. In fact, due to the ubiquitous nature of IR exposure, cellular systems have developed well-orchestrated DNA repair molecular pathways, highly specialized and differentiated to deal with the several classes or IR-induced lesions, a machinery collectively known as DNA damage response (DDR) [3]. Repair capability depends by the sheer amount of initially induced DNA damage, which is a function of the absorbed radiation dose, but also by the quality of radiation, i.e., the ionization density along radiation tracks.

**Figure 1:**
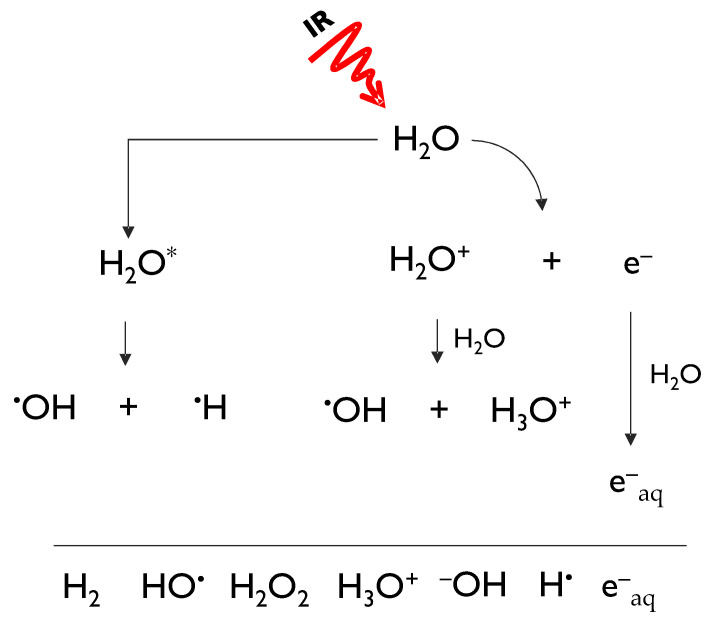
Hydrated electrons (e−_aq_), and radical and molecular species generated during water hydrolysis.

Obviously, naturally occurring background radiation is not the only source of human exposure to IR [4]. At about the same time light was shed on the laws governing the process of natural radioactive decays, it became evident that IR could be artificially generated. The impact that the discovery of X-rays by Wilhelm Conrad Roentgen in 1895 has had on many aspects of human health is still reverberating nowadays, as IR is widely used in both the diagnosis and treatment of diseases [5]. Insofar as the therapeutic use of IR is concerned, the very same DNA-damaging action by IR that classifies it as a hazard to human health is exploited by its ability to eradicate cancer cells though radiotherapy. Much is known about the way IR brings about its biological effects thanks to extensive radiobiological research that has unveiled basic mechanisms. To this aim, it is useful to classify IR as indirect and direct, based on the way energy is released in the (biological) matter. Photons, such as X-rays and γ-rays, and neutrons act indirectly, requiring a two-step action before causing any potentially biological relevant damage. In fact, photons interact with the atom shell-containing electrons thereby generating secondary fast-moving electrons, which in turn cause further ionizations, with the emission of slower electrons; neutrons interact with the nuclei of the traversed material, giving rise to charged particles such as protons and heavier nuclei [5]. Charged particles, instead, lose energy directly through Coulomb interactions, producing the above-mentioned ionizing tracks along their penetration depth [5]. Biological effects related to IR, whether caused by direct or indirect radiations, are classified into direct and indirect, too. In the first case chemical alteration of biomolecules are formed during the physico-chemical stage that temporally precedes the actual biological stage. Instead, they are indirect when they are the result of radiation products, such as free radicals generated by water radiolysis. Indirectly generated DNA damage is the only form of damage whose amount can be modulated by concomitant agents, such as antioxidant compounds. In fact, when ionizing radiation passes through water, it leads to a number of ionic and excited states that further decompose or recombine to give hydrated electrons (e−_aq_) and reactive species, including hydrogen radical (H^●^), hydroxyl radical (OH^●^), hydrogen peroxide (H_2_O_2_), oxygen (O_2_), hydrogen (H_2_), and hydroperoxyl radical (HO_2_^●^) (Figure 1) [6].

DNA DSBs are universally regarded as the most deleterious IR-induced lesion [7]. DDR can lead to cell-cycle checkpoint activation, hence cell cycle delay/arrest, in the attempt to increase time for repair. Irrespective of which mechanism the cell uses, un-/mis-repaired DSBs can lead to cell death through several pathways (e.g., mitotic failure, apoptosis), typically arising at first mitosis post-irradiation or after a few cell cycles from exposure. This is the aim of curative radiotherapy. However, failure in correct restoration of the DBS can result in rearrangements of genetic material (e.g., chromosome aberrations, micronuclei), which, if transmissible through cell division, can cause late-arising effects, leading to generalised genomic instability, and hence to an increase in the risk of malignant transformation [8].

Conventional radiotherapy by high-energy photon or electron beams is a mainstay of modern cancer treatment, with an estimated 50% of cancer patients receiving it alone or in combination with other modalities worldwide [9]. Although several improvements have been achieved in dose delivery accuracy, the mitigation of noncancerous normal tissue toxicity remains of crucial importance because of the above-mentioned secondary cancer risk affecting the unavoidably exposed normal tissues and/or organs at risk. Since photons are mainly characterized by the indirect mode of action, the amount of damage they produce during the physical stage, can be modulated during the chemical stage prior to damage fixation and before the onset of the biologically driven DDR. Therefore, modifiers/protectors can be utilized to selectively benefit normal tissues, delivering further minimal toxicity [10]. In this context, several compounds have been described, but only amifostine, the S-phospho derivative of 2-[(3-aminopropyl)amino]ethanethiol, is approved as clinical radiation protector [11]. Other thiol-containing compounds, beyond nitroxides with superoxide dismutase (SOD)-like activity, hormone analogues, antibiotics, and phytochemicals, have been investigated as radioprotectors, whereas immunomodulators, probiotics, statins are explored as mitigator agents [12]. Specialized natural compounds are playing a key role in preclinical and clinical research, thanks to their anti-oxidant and anti-inflammatory efficacy that identifies them as promising agents in the field of radioprotection and radiomitigation.

## 2. Radioprotection: A Valuable Approach to Counteract Radiation Exposure

Although radiotherapy is one of the most effective strategy to treat cancer, the normal-tissue response is the limiting factor for the total dose that can be safely administered to achieve tumour local control, hence reducing the chances of cure, while acute and chronic toxicities may lead to an overall poor patient’s life quality. Therefore, an urgent need in protecting normal cells is actively claimed. In this context, technological improvements in IR delivery and accuracy are ongoing, whereas radiomodulating agents are considered a valuable alternative to decrease toxicity to normal tissues. This is not an emerging issue so much so that the IR research program of the National Cancer Institute classified, according to administration timing, agents with IR protective properties in three categories: (a) protection, (b) mitigation, and (c) therapeutic agents [13]. Analogously, the European Commission has paid and continues to pay great attention to radiation protection, generally addressing new research findings with potential policy and/or regulatory implications [14].

Radioprotectors and radiomitigators are valuable modulator agents. Their delivery precedes or occurs simultaneously with the radiation administration and is prompt to reduce or ameliorate normal tissue toxicity. The latter could take advantage of therapeutic compounds, when an adverse effect is established, acting after irradiation as palliation or support [15].

**Figure 2:**
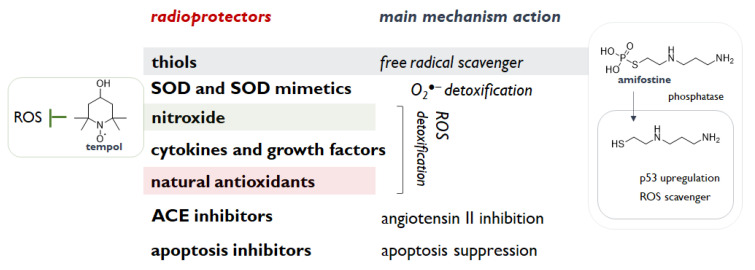
Radioprotectors’ categories and main ascribed mechanisms of action.

All these compounds need to share some functional features such as the ability to interrupt or slow down the overproduction of reactive species, which can perpetuate indefinitely the IR injury affecting different cell activities and signalling pathways. Indeed, reactive oxygen and nitrogen species reinforce IR cytotoxicity. Counteracting the onset of oxidative stress conditions prevents structural and functional disruption of nucleic acids, proteins and lipids, and a series of processes (e.g., mitochondrial depolarization), which irreversibly lead to cell death [16]. IR-induced genomic instability is the main target to encompass, as mutations, gene amplification and other cytogenetic rearrangements could be also after the initial insult [17]. Cells adaptatively respond to IR by activating the Nrf2-ARE antioxidant defence [18], which is constituted by enzymatic and non-enzymatic compounds, and can benefit radioprotectors with the aim to evade free radicals, remove IR-induced toxic substances, and overall to intensify the repair and recovery processes [19]. Thus, compounds able to scavenge these species or enhance antioxidant enzymes (e.g., superoxide dismutase, catalase, and glutathione peroxidase) should properly investigated. In this context, thiols, thanks to their ability to scavenge hydroxyl radical, protect DNA, which provides, mostly in hypoxic condition, harmful DNA radicals, likely responsible for radiation lethality [10]. Furthermore, thiols are observed to prevent the oxidation of membrane phospholipids, and to modulate cell recovery and stress responses. Cysteine and cysteamine are sulfhydryl amines, and other aminothiol analogues/derivatives appeared to be radioprotective, but their side effects advised against clinical use, with the exception of the aminothiol amifostine (WR-2721) [10]. Among the deeply studied species, nitroxides are also of great interest thanks to their single-electron redox cycle ability. In particular, as a pleiotropic intracellular antioxidant, tempol was observed to reduce the incidence of radiation-induced second malignancies [20]. Indeed, tempol (4-hydroxy-2,2,6,6-tetramethylpiperidine-N-oxyl) was also shown to act as a SOD mimetic, whereas the radioprotective properties of the SOD isoforms (Cu, Zn SOD, Mn SOD and extracellular SOD) are highlighted as useful agents for their O_2_^−^^●^ scavenging efficacy in cytosol, mitochondrion, and extracellular space, respectively, and their-catalysed dismutation to H_2_O_2_ and O_2_. Other categories of radioprotective agent are cytokines and growth factors, including IL-1, TNF-α, G-CSF, GM-CSF, and erythropoietin, and angiotensin-converting enzyme inhibitors (Figure 2). These latter compounds, routinely prescribed for hypertension treatment, were observed to ameliorate radiation side effects in kidney, lung and brain and to interfere with TGF-β pathway, which could contribute to radiation-induced fibrosis [10]. Among these agents, palifermin, a recombinant N-terminal truncated form of keratinocyte growth factor, was firstly approved for the treatment of oral mucositis induced by chemo- and radio-therapy [21]. Inhibitors of PUMA (p53 Up-regulated Modulator of Apoptosis) and radiation-induced apoptosis were also investigated. PUMA inhibitors (PUMAi) are designed to inhibit PUMA-dependent and radiation-induced apoptosis and to avoid or alleviate intestinal damage and apoptosis induced by inflammatory cytokines, ROS (reactive oxygen species), or cancer therapy [22].

Natural antioxidants also find growing interest. Vitamins (A, C, and E), L-selenomethionine, N-acetylcysteine, glutathione, and coenzyme Q-10 are suggested to be effective against radiation injury [23], while several dietary phytochemicals appeared to act as radioprotectors for normal cells, and radiosensitizers for tumour cells in a fascinating scenario. Melatonin, which is secreted by the pineal gland in the brain, from lymphocytes, the retina, and gastrointestinal system, is one of the most studied natural occurring compounds. It directly scavenges ROS species, inhibits ROS forming enzymes, and activates DNA repair enzymes [24].

The lack of harmful toxicity, together with their appreciable antioxidant and immunostimulant activities, make specialized metabolites from plants an endless reservoir of radioprotective compounds. Polyphenols, through their intrinsic antioxidant capability, are able to reduce inflammation, protecting both immune and hematopoietic systems, and preserving DNA. In particular, flavonoids, such as rutin and baicalein, the isoflavonoid genistein, and the stilbene resveratrol, represent compounds strictly related from a biosynthetic point of view and are promising radioprotective candidates. The poor bioavailability of these substance encourages new administration forms, and the phytochemical research for the discovery of new compounds from hopeful plant extracts. An update of the radioprotective natural molecules and an examination of the plant extracts enriched in these constituents are provided below.

## 3. Phenols and Polyphenols: Are They a Valuable Radioprotective Strategy?

The failure of synthetic compounds as effective radioprotectors allowed researchers to focus on natural substances and their radioprotective efficacy, and several botanicals, which could be less expensive than synthetic ones, have been screened for their radioprotective activity [25].

Free radical scavenging, anti-inflammation, facilitation of repair activity, regeneration of hematopoietic cells, are the main mechanisms attributable to natural radioprotectors (Figure 2). In particular, since most of the IR damage in the conventional radiotherapy setting arises from the interaction of IR-induced free radicals with biomolecules, natural substances, such as curcumin, chlorogenic acids, and different flavonoids, being able to destroy free radicals or prevent their formation, could serve as radioprotectors [26]. Thus, the use of radioprotectors for protecting normal tissue and of radiosensitizer for augmenting cancerous tissue response appeared to be innovatively maximized in a toxicity-free nutraceutical approach based on polyphenols. These natural compounds summarize the concept of an ideal protector, as, based on their dose-dependent antioxidant/pro-oxidant efficacy, could provide a high degree of protection to normal tissues, with little or no protection to tumor cells. Moreover, plant-derived polyphenols have gained a lot of attention in the long-standing quest for intrinsically low-toxic radiosensitizing drugs.

The attractive double-edged potential of pure polyphenols or polyphenol-enriched extracts provided good evidence on their possible radiomodulating action, and the polyphenol dual ability to act as both radiosensitizing and radioprotective agents would arguably hold pre-clinical significance, and, more generally, bear a significant impact on the prognosis of tumors refractory to radiation treatment.

### 3.1. Flavonoids: The Double-Edged Sword in Radioprotection

**Figure 3:**
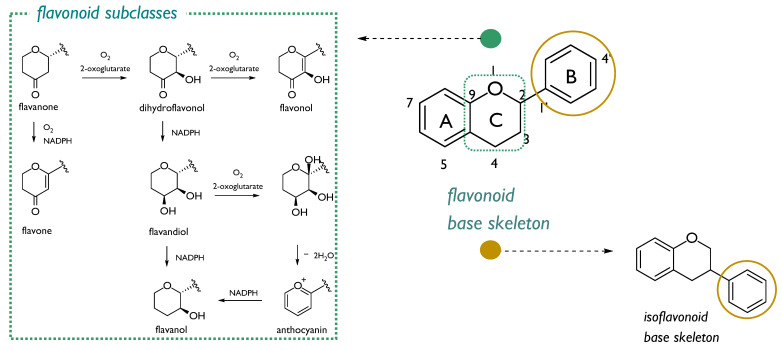
Flavonoid base skeleton and structural modifications of ring C which led to different flavonoid subclasses (green box). Isofavonoids are from the 1,2-aryl shift (yellow circle).

Over the last twenty years, the interest in radioprotective flavonoids is ongoing. These plant metabolites, commonly biosynthesized as defense compounds against UV-radiation, and other environmental stresses through chalcone precursors, are structurally characterized by a 15-carbons skeleton, consisting in two benzene rings (A and B) linked through a heterocyclic pyrone C-ring atoms. A high degree of hydroxylation, substitution, and polymerization is in flavonoid class, which consists in seven sub-classes: flavanones, dihydroflavonols, flavonols, flavones, flavandiols, anthocyanins, and catechins (Figure 3). Isoflavonoids form a well-separated flavonoid subclass, as these compounds showed a structural variant feature in which the B-aromatic ring is located at C3 carbon (Figure 3) [27]. Investigations aimed to explore structural features involved in radioprotection highlighted that some flavonoid compounds (mainly those sharing the keto group conjugated to aromatic rings) could be valid agents, because protection is related to their ability to inhibit energy transfer processes and to stabilize redox processes in irradiated cells [28].

**Figure 4:**
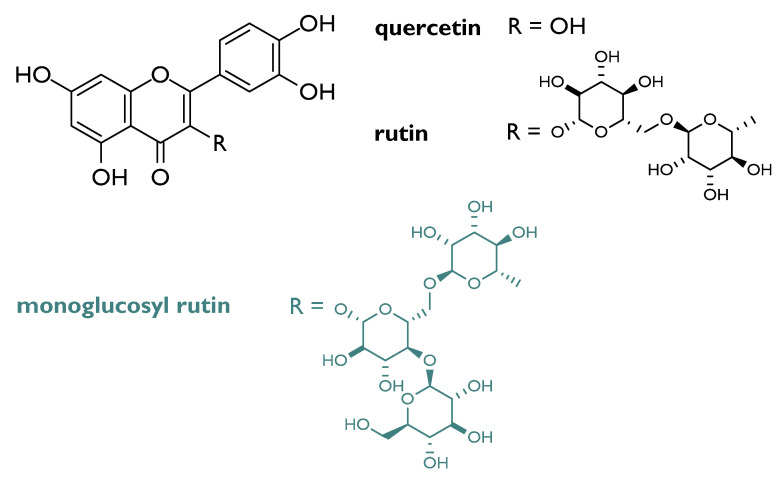
Chemical structures of quercetin, rutin and its monoglucosyl derivative.

Flavonols appeared the most valuable compounds, although glycosylation on C-3 carbon affects the reactivity, based on the saccharidic moiety identity. In fact, it was observed that flavonol glucosides decrease in reactivity when the sugar forms two intramolecular H-bonds. Furthermore, based on aglycone substitution, more phenolic functions are present, more the compound is active [28]. Among flavonol compounds, rutin (3,3′,4′,5,7-pentahydroxyflavone-3-rhamnoglucoside; Figure 4), abundant in passion flower, buckwheat, tea, and apple, is broadly investigated for its radioprotective action. Cell culture assays data highlighted its ability to protect from radiation-induced oxidative DNA damage in cells (e.g., V79).

Rutin daily supplementation, as well as that of its aglycone, namely quercetin, reduced the frequency of micronucleated reticulocytes in the peripheral blood of irradiated mouse. Combining podophyllotoxin and rutin in G-003 formulation, it was found a significant protection of the mice hematopoietic, gastrointestinal, and respiratory systems against lethal radiation dose [29,30,31]. The monoglucosyl rutin, ad hoc semi-synthetized to overcome quercetin and rutin insolubility in aqueous media, was proved to be effective towards CHO 10B2 cells, being able to increase the survival of IR-treated cells at doses greater than 2 Gy [32]. Indeed, the in vitro DNA double-strand breaks analysis, carried out on different flavonoid aglycones and glycosides, evidenced that, although quercetin derivatives reduced DNA double-strand breaks at a concentration equal to 10 μM, they low bioavailability could affect their efficacy in vivo [33].

Protection from DNA damage in γ-irradiated white blood cells [34], leucocytes [35] was also ascertained for quercetin and its enriched natural matrix propolis, so much so that further investigation in animal models was performed. In particular, the protective effect of an aqueous propolis extract against intestinal radiation damage was also evidenced in rats exposed to a γ-radiation dose of 8 Gy, able to induce intestinal mucositis [36], whereas a propolis methanolic fraction, with high content in both simple phenols and flavonoids, lowered total protein carbonyl content in UV-treated HaCat cells [37].

**Figure 5:**
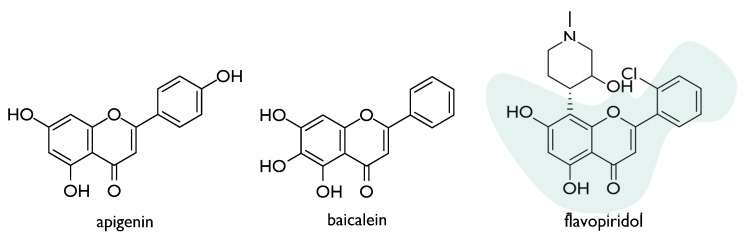
Chemical structures of radioprotective natural (apigenin and baicalein) and semisynthetic (flavopiridol) flavones.

The radioprotective effect of flavones, such as apigenin and baicalein, was also deeply investigated (Figure 5). Apigenin, widely distributed in the leaves and stems of dietary vegetables and fruits, dose-dependently induced micronuclei in human lymphocytes treated in vitro, also suppressing adverse effects of ionizing radiation [38]. The compound appeared to exert immunostimulatory effect in vivo, thus mitigating radiation-induced hematological alterations. This outcome could be due to its ability to trigger the endogenous antioxidant status [39]. Recently, apigenin, intraperitoneally administered at a dose level equal to 15 mg/kg body weight, was found to slow down radiation-induced gastrointestinal damage in whole-body irradiated Swiss albino mice. In particular, the restoration of intestinal crypt-villus architecture appeared to occur following apigenin pre-treatment, as well as the inhibition of the radiation-induced activation of NF-κB expression in the gastrointestinal tissue [40].

**Figure 6:**
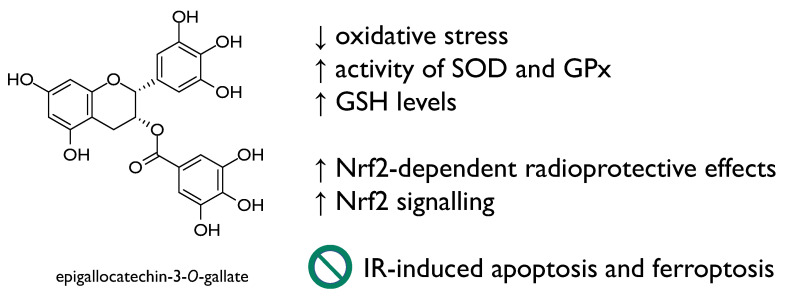
Epigallocatechin-3-*O*-gallate radioprotective mechanisms of action.

Naringin, a flavanone-7-*O*-glycoside from Citrus species, also showed inhibitory effect towards IR-induced inflammation. The NF-κB suppression defined the alteration of pro-inflammatory factors. Moreover, naringin reinforced the intracellular defense mechanisms, through the preservation of endogenous antioxidants [41]. The oxidative stress inhibitory activity was also linked to the release of inflammatory cytokines by inducing Nrf2 activation, a common feature of other flavonoid compounds, such as naringenin and epigallocatechin-3-*O*-gallate (Figure 6) [42]. Moreover, regarding flavone compounds, baicalein (5,6,7-trihydroxyflavone), originally isolated from the dried roots of *Scutellaria baicalensis* and *Scutellaria lateriflora*, elicited pleiotropic activity that allowed it to protect mouse splenic lymphocytes against IR-induced cell death through its ability to suppress MKP3 and activate ERK. This is in line with mitigation of radiation-induced hematopoietic injury [43]. Recently, baicalein, administered intraperitoneally with 100 mg/kg in C57BL/6J mice, rebalanced IR-altered gut microbial composition, ameliorating intestinal structure. It down-regulated the expression of pro-apoptotic proteins (e.g., p53, caspase-3, caspase-8 and Bax), also recovering IR-induced hematopoietic dysfunction [44]. Baicalein was reported as a potent radioprotector at the concentration of 5–50 μM [45] and impacts on the NF-κB-mediated inflammatory response [46].

Growing evidence suggests the potential benefit from green tea flavanols. Early studies supported the hypothesis of anti-genotoxic efficacy in human lymphocytes [47], and the overall prevention against ultraviolet radiation-induced DNA damage [48].

Epigallocatechin-3-*O*-gallate (Figure 6), the main polyphenol in green tea, due to its antioxidant activity and the efficacy in ameliorating many oxidative stress-related diseases, is of wide interest. It promoted Nrf2-dependent radioprotective effects and Nrf2 signalling, in turn, and was found to repress IR-induced apoptosis and ferroptosis, ameliorating intestinal injury induced by total body irradiation in male C57 BL/6 J mice [49]. The radioprotection of EGCG was studied through a model of oxidative damage in ^60^Coγ radiation mice, and data acquired evidenced the ability of the compound to enhance the activity of enzymatic antioxidants, such as superoxide dismutase and glutathione peroxidase, as well as glutathione levels [50].

Soy isoflavones mitigate vascular damage and inflammation related to lung cancer radiotherapy [51]. Genistein, a main soy isoflavone with phytoestrogen activity, enjoy dual action in radiotherapy; first, it can protect L-02 cells against radiation damage via inhibition of apoptosis, alleviation of DNA damage and chromosome aberration, down-regulation of GRP78 and up-regulation of HERP, HUS1 and hHR23A at low concentration (1.5 µM). Secondly, at high concentration (20 µM) indicate radio-sensitizing properties through the promotion of apoptosis and chromosome aberration, impairment of DNA repair, up-regulation of GRP78, and down-regulation of HUS1, SIRT1, RAD17, RAD51 and RNF8 [52]. Indeed, recently, it was observed that genistein is able to augment the radiosensitivity of hepatoblastoma cells by inducing G2/M arrest and apoptosis [53]. Administration of the genistein also demonstrated providing protection against acute radiation injury at non-toxic doses [54]. Several evidences underline genistein-induced radioprotection for the hematopoietic acute radiation injury, and the ability of the compound to act as a selective estrogen receptor β-agonist was also explored, as it is involved in its radioprotective mechanism of action [55]. The up-regulation of ER-β and FOXL-2 by genistein, with associated downregulation of TGF-β expression was implied also in reversing radiotherapy-induced premature ovarian failure [56]. Soy isoflavones are overall prone to modify clinical responses to RT, acting both with radiosensitizing and radioprotective effects. In preclinical orthotopic models of prostate cancer, renal cell carcinoma and non-small cell lung cancer, it was observed that soy isoflavones targeted signaling survival pathways radiation-upregulated, such as DNA repair and transcription factors, finally driving cancer cells to death [57].

The interest in flavonoids as radiomodulators also led to screen the properties of semisynthetic drugs, such as flavopiridol (Figure 5). This compound, also known as alvocidib, is a flavone derivative that was developed by Sanofi-Aventis, based on a flavonoid derived from the Indian indigenous plant *Dysoxylum binectariferum*. Flavopiridol, structurally based on flavonoid (2-cholorophenyl-4-one) and an alkaloid (1-methylpiperadine) moieties, is a CDK inhibitor that exhibited potent inhibition of CDK1, 2, 4, 6, 7, and 9. It was observed that the compound acts to inhibit and/or repair sublethal damage as well as the repair of DNA double-strand breakage followed by radiation therapy in malignant tumours. Indeed, it might enhance the cytotoxic effect of radiation in radioresistant tumour cells through p53 dysfunction or Bcl-2 overexpression [58].

Reducing the harmful effects of UV radiation is an important issue to pursue, as UVB (290–320 nm) could destroy the integrity of the skin causing epidermal cell apoptosis, potentially even leading to skin cancer. Thus, radioprotective compounds need to be explored and anthocyanins appeared valuable candidates. In particular, the protecting effect of cyanidin-3-*O*-glucoside against UVB-induced damage, one of the harmful factors for the benefit of human skin, to human HaCaT keratinocytes. The anthocyanin was able to decrease intracellularly reactive oxygen species, as well as phospho-p53 and phospho-ATM/ATR levels, and the expression of anti-apoptotic protein B-cell lymphoma 2 [59]. Indeed, it was also demonstrated that cyanidin-3-*O*-glucoside suppressed COX-2 expression by interaction with the MAPK and Akt signalling pathways [60]. Excess ultraviolet (UV) radiation causes numerous forms of skin damage. The encapsulation of cyanidin-3-*O*-glucoside in chitosan nanoparticles provided evidence for the efficacy of the formulation to effectively reduce UVB-induced epidermal damage through the p53-mediated apoptosis signalling pathway [61].

### 3.2. Other Phenols and Polyphenols with Radioprotective Efficacy

Non-flavonoid compounds were also analyzed in pure form or in mixture. Simple phenols, such as vanillin, were screened for their radioprotective activity.

The compound 4-hydroxy-3-methoxybenzaldehyde, better known as vanillin, widely used as food flavoring agent, was previously investigated as able to counteract γ-radiation-induced DNA damage in plasmid pBR322, human and mouse peripheral blood leucocytes and splenic lymphocytes. The positive action was ascribed to its radical scavenging capability, as well as to the modulation of DNA repair [62]. The antioxidant power of vanillin also includes its ability to act as lipoperoxidant. Moreover, the compound exhibits anti-mutagenic effects being able to inhibit X-ray and UV-induced single-strand DNA breaks, chromosomal breaks, and DNA crosslinking. Furthermore, although it was shown to also favor DNA ligation, repair and replication, its clinical use is limited by the low in vivo activity. This finding promoted the synthesis of its derivative, VND3207, which appeared to be, in a preclinical screening, radioprotective towards radiation-induced intestinal injury [63]. In this context, it was observed that radioprotection is due also, beyond the antiradical properties of the compound, to the modulation of activated p53 levels in intestinal epithelial cells. Recently, Li et al. [64] showed that treatment with VND3207 can enhance the expression of the catalytic subunit of the DNA-dependent protein kinase (DNA-PKcs) in human lymphoblastoid cells with or without γ-irradiation. Also in this case, the activity of the enzyme consisted in DNA double-strand breakage repair.

Hydroxycinnamic acids, commonly found in fruits, vegetables, and beverages, and structurally characterized by a phenylpropenoid skeleton deriving from the deamination of phenylalanine and tyrosine in plants, could be mirrored in radioprotection. In recent times, caffeic acid was found to ameliorate premature senescence of hematopoietic stem cells, due to its antioxidant capacity. In fact, senescence is mediated by ROS overproduction. On the other hand, caffeic acid can act as pro-apoptotic agent in colon cancer cells [65]. Ferulic acid was also hypothesized to be effective against accident or intentional exposures to ionizing radiation, and the repair of DNA was experimented to take place at a faster rate in ferulic acid treated mice [66].

Among hydroxycinnamoyl derivatives, chlorogenic acid prevented genomic instability induced by X-ray irradiation in non-tumorigenic human blood lymphocytes [67]. Furthermore, the treatment with this depside, at dose level equal to 200 mg/kg, one hour prior to irradiation with high doses of γ-radiation, favoured the animal survival [68].

The radioprotective ability of caffeic acid phenethyl esters, which are abundant in propolis, also was studied and it involves in prevention of oxidative and nitrosative damages induced by radiation [69]. In another study, caffeic acid phenethyl ester was found to act both as radioprotector and radiosensitizer, meaning that it can modulate the radiation response by following different mechanisms depending on the tissue type [70].

Rosmarinic acid, a depside of caffeic acid and 3,4-dihydroxyphenyl lactic acid, promoted, when administrated at a dose of 100 mg/kg, the recovery of peripheral blood cells in irradiated mice [71]. Its capability was also compared to that exerted by carnosic acid and carnosol, two aromatic diterpenes, endowed with antioxidative and antimicrobial properties, equally isolated from rosemary herb. Indeed, the radioprotective effects against γ-irradiation was in the order carnosic acid > rosmarinic acid ≥ carnosol [72].

Radioprotection research pays peculiar attention to curcumin, whose antioxidant and anti-inflammatory properties are well-known to target multiple signalling molecules [73].

The diferuloylmethane was demonstrated to ameliorate radiation-induced pulmonary fibrosis [74]. Its effect was through up-regulation of the cytoprotective heme oxygenase 1. Furthermore, as oxidative stress is involved in radiation pneumonopathy, the inhibition of γ-radiation-induced reactive oxygen species in murine lung primary cells was detected. The preventive curcumin outcome was also in ileum goblet cells [75], and on *Drosophila melanogaster* lifespan [76]. Inhibition of the transcription factor NF-κB is the main mode of action of curcumin and is involved also in curcumin-based radiosensitization [77].

Recent findings suggest the capability of a pre-treatment with curcumin to prevent radiotherapy-induced oxidative injury to the skin, through enhancing CAT, SOD, and GSH-Px [78]. Indeed, the low bioavailability of curcumin, due to poor absorption, rapid metabolism, and rapid systemic elimination [79], was carefully taken into account when approaches aimed at preserving its functionality were investigated. In this context, there was the formulation of nanoscale curcumin-encapsulated liposomes [80], or the design of curcumin conjugated albumin-based nanoparticles. In particular, the improvement of radioprotection of conjugated albumin-based nanoparticles was estimated in HHF-2 cells X-ray irradiated, finding that nanoparticles with curcumin at 50 µg/mL induced a 2.3-fold increase in cell viability in respect to cells which underwent only X-ray irradiation [81].

Table 1 summarizes literature data for all phenolic and polyphenolic compounds taken into account in the above discussion, ordered alphabetically, with details about the studied model (in vitro or in vivo), the used dose, and the main protective effect(s).

## 4. Bioactive Plant Extracts in Radioprotection: A Still Undervalued Topic

Plant extracts possess infinite therapeutics, such as anticancer, antioxidant, antimicrobial, anti-inflammatory, and analgesic. According to the World Health Organization (WHO), about 80% of people worldwide use traditional medicine for primary healthcare needs [82]. There are nearly 20,000 medicinal plants in 91 countries which contain a wide range of substances that can be used to different therapeutic purposes. Different plant extracts rich in phenols and polyphenols and/or other specialized metabolites (e.g., carotenoids, sulphur compounds) were screened for their radioprotective effects, and potential mechanism of actions were proposed.

Indeed, although promising efficacy was suggested, literature data often lack detailed chemical composition analyses and standardization, compromising data reproducibility.

The activities in radiation protection as evaluated in extracts obtained from medicinal plants, widely used in complementary and alternative medicine, or from food plants are reported below. Alcoholic extract of the plant *Ageratum conyzoides* was able to protect mortality in mice exposed to 10 Gy of γ-radiation. Accordingly, up to a dose of 3000 mg kg^−1^ was attributed to non-toxic concentration, suggesting that the radioprotection afforded by *Ageratum conyzoides* may be in part due to the scavenging of reactive oxygen species induced by ionizing radiation [83].

The investigation on extracts from five medicinal plants including *Adhatoda vasica*, *Amaranthus paniculatus*, *Brassica compestris*, *Mentha piperita* and *Spirulina fusiformis* indicated that the antioxidant capacities of these plant extracts can be responsible for radioprotective capacities [84]. According to this study, major chemical constituents play a key role in radiation protection. These compounds were vesicine, vesicinone, betaine, vitamin C, β-carotene, and vasakin in *Adhatoda vasica*; proteins, vitamins (C and E), provitamin A, and riboflavin in *Amaranthus paniculatus*; allyl isothiocyanate, glucosinolates, indoles in *Brassica compestris*, and proteins, natural vitamins (β-carotene), and SOD in *Spirulina fusiformis*.

Several pathways of protection against ionizing radiation have been suggested in mammalian cells [85]. These mechanisms involve free radical-scavenging through the inhibition of reactive oxygen species, as well as hydrogen atom donation [86,87]. It can be concluded that phenolic compounds due to their antioxidant activities can act as free radical scavenger and, likewise radioprotectors [84]. Recently, an aqueous extract from a Southern Italian cherry cultivar, constituted by chlorogenic acids and flavonoids together with simple carbohydrates and polyols, proved to exert a radiomodulating behaviour against SH-SY5Y neuroblastoma cell line. In fact, at low doses it acted as a radioprotector agent, whereas at high doses it enhanced cytotoxic effects due to radiation exposure [88].

*Olea europaea* leaf is a rich source of phenols and polyphenols, whose radioprotective potential was marginally investigated in pre-UV and post-UV treatments [89]. Anticlastogenic and antiradical activities of an olive leaf extract, constituted by 24.5% in oleuropein, 1.5% in hydroxytyrosol and by almost 3% in flavone-7-glucosides, and 1% in verbascoside, was found. The effects of pure oleuropein on radiation response in nasopharyngeal carcinoma were also determined [90]. Oleanolic acid and ursolic acid, two triterpene acids from olive fruit and other dietary products, could inhibit the tumor growth and modify hematopoiesis stem cells (HSCs) after irradiation [91]. In addition, anti-tumor activity was perforemed by the interplay of oleanolic acid and ursolic acid, so that they might partially act as anti-cancer agents and, furthermore, decrease damages occurring on hematopoietic tissue after radiotherapy [91,92]. The radioprotection by apple polyphenols was also investigated through in vitro studies mainly aiming to clarify the polyphenols’ ability to scavenge free radicals [93].

*Rheum palmatum* L., and its main compound emodin (6-methyl-1,3,8-trihydroxyanthraquinone), were radioprotective against γ-rays. Emodin’s mechanism of action is somehow that the levels of total thiols such as glutathione and lipid peroxidation products have been decreased. Furthermore, measuring of tongue antioxidant enzymes, glutathione peroxidase, glutathione-S-transferase, γ-glutamyl transferase, and glucose-6-phosphatase, revealed the amelioration of the levels of cellular thiols and antioxidant enzymes in serum of gamma irradiated diabetic mice with emodin treatment [94].

The ethanolic extract of leaves of *Adhatoda vasica* L. Nees, a well-known plant drug in Ayurvedic and Unani medicine, showed a significant decrease in acid phosphatase level and, on the contrary, an increase in alkaline phosphatase level. Pre-treatment with Adhatoda also significantly demonstrated the prevention of radiation-induced chromosomal damage in bone marrow cells. However, it remains the necessity of mechanistic studies for its radioprotective effects as well as major ingredients in the extract thereof [95]. Leaf extract of *Adhatoda vasica* was also reported for the protective role in spleen of Swiss albino mice exposed to 6 Gy γ-radiation [96].

Radioprotective efficacy of *Amaranthus paniculatus* leaf extract have been reported [97,98]. The oral administration of *A. paniculatus* extract at 800 mg/kg body weight of Swiss albino mice for 15 consecutive days before exposure to gamma ray showed the increased endogenous spleen colonies and the spleen weight without any side effect or toxicity. The modulation of glutathione as well as lipid peroxidation were other results thereof [97].

Lamiaceae family is attributed to have radioprotector ability, with mechanisms including mainly free radicals scavenging, DNA damage protection, decreasing of lipid peroxidation and enhancing of glutathione, superoxide dismutase, catalase, and alkaline phosphatase enzymes activity levels [99]. For instance, *Mentha piperita* is a plant belonging to this family, containing eugenol, caffeic acid, rosmarinic acid, and α-tocopherol that play a key role for anti-cancer and radioprotective properties [84,100].

Remedial and pharmacological properties of aromatic plants have been reported. Amongst, essential oils are used for different aspects such as in cosmetics, fragrance, pesticides, and beverages. Since essential oils are known for their antioxidant activities and free radical scavenging, they can be also considered as radioprotectors. *Ageratum conyzoides* has shown DPPH radicals scavenging, likewise its radioprotective role with the dose of 75 mg/kg for 6–11 Gray on mice. Active agents in this plant are polyoxygenated flavonoids, triterpenes (friedelin), sterols β-sitosterol and stigmasterol, and alkaloids (lycopsamine and echinatine) [83].

*Alium cepa*, *Alium sativum* are two plants from Liliaceae family which enjoy antioxidant, antihypertensive and antihyperglycemic [101]. Alk(en)yl thiosulfates from onions and garlic markedly reduced damage in rat hepatoma H4IIE cells and mouse lymphoma L5178Y cells treated with 10 Gy of X-ray irradiation [102].

Recently, the radioprotective efficacy of an hydroalcoholic extract of *Pterocarpus santalinus*, a small-to-medium–sized deciduous tree belonging to the Fabaceae family [103], was verified in BALB/c mice exposed to γ-radiation. The redox homoeostasis corrupted by the radiation was ameliorated following the treatment with the extract, which probably occurred via the upregulation of Nrf2, HO-1, and GPX-1p. The UHPLC-HRMS/MS analysis of the extract highlighted its diversity in santolins, beyond other phenols and flavonoid compounds [104]. Furthermore, the *Pterocarpus santalinus* hydroalcoholic extract (PSHE) was non-toxic, and when RAW264.7 macrophages were pretreated with it, a significant inhibition of LPS-induced pro-inflammatory cytokines IL-6, and TNF-α production was observed. Polyphenols from medicinal plants, such as *Sanguisorba officinalis* and *Erigeron canadens*, were found to be able to decrease irradiation-induced oxidative stress in normal lymphocytes using ROS mechanisms, acting as a radiation modifier for normal cells [105]. An extract from *Lonicera caerulea* var. *edulis*, rich in anthocyanins, and intragastrically administered to mice once a day, prior to 5 Gy whole body ^60^Coγ radiation, was effective to slow down the levels of malondialdehyde, while increasing superoxide dismutase and glutathione peroxidase activities and glutathione GSH content in the liver [106].

## 5. Conclusions

Over the last two decades, secondary metabolites in plants have been broadly considered for their therapeutic attributes as radioprotectors. Indeed, the lower toxicity and cost of natural products appear two key factors that push the research to deepen the understanding of the mechanism of action of these substances. Thus, several studies are found in literature focused on their role in counteracting IR-induced damage. Pure compounds or plant extracts showing free radical scavenging activity, anti-lipoperoxidant, and reducing properties are of predominant interest and are prone to intervene in DNA repair or in restoring chromosomal damages.

In particular, (poly)phenols could represent a valuable alternative to synthetic compounds to be used as radioprotective agents. In fact, based on their dose-dependent antioxidant/pro-oxidant efficacy, they could provide protection to normal cells, with little or no protection to tumor cells resistant to radiotherapy. Thus, they provide good evidence regarding a possible radiomodulating action.

Furthermore, considering the dietary nature of the majority of matrices investigated, another great advantage could derive from the suitability of oral administration that could be optimal during radiotherapy. However, with few exceptions, the data available to date remain fragmentary and are mostly the result of in vitro studies which, while deepening the chemical and biological knowledge of the molecules, do not find comfort in preclinical and/or clinical studies, or preclinical studies in which, especially when evaluating the bioactivity of a plant extract, its chemical composition is not taken into account.

## Figures and Tables

**Table 1:** Radioprotective properties of natural phenols and polyphenols herein described (BW = body weight; i.p. = intraperitonially; s.c. = single subcutaneous; i.m. = intramuscular; i.g. = intragastrical).

Compound	Studied Model	Dose	Main Protective Effects	Ref.
Apigenin	Human lymphocytes	Up to 25 μg/mL	Protection from ^137^Cs gamma rays-induced chromosome aberrations	[38]
	Swiss albino mice	15 mg/kg BW i.p. for 7 consecutive days	Immunostimulatory effect and mitigation of radiation-induced hematological alterations	[39]
	Swiss albino mice	15 mg/kg BW i.p. for 7 days	Restoration of intestinal crypt-villus architecture Inhibition of the radiation-induced activation of NF-κB expression in the gastrointestinal tissue	[40]
Baicalein	Swiss and C57BL/6 male mice	10 mg/kg BW for 3 days	Activation of the target molecules ERK and Nrf-2 both in vitro and in vivo	[43]
	Murine T cell lymphoma cells (EL4)	5–100 μM		
	Swiss albino mice	150 mg/kg BW	Protection from DNA damage Reduction of radiation-induced damage to mice bone marrow cells	[45]
	Human white blood cells	Up to 50 μM	Dose dependent inhibition of DNA strand breaks	
	C57BL/6 mice	5 mg/kg BW for 3 days	Protection against NFκB-mediated inflammatory response through MAPKs and the Akt pathway Up-regulation of FOXO activation, catalase and SOD activities	[46]
Caffeic acid	Mouse colon cancer (CT-26), human liver cancer (HepG2) and human breast cancer (MCF-7) cells lines	1–3 mM	Induction of apoptosis of colon cancer cell	[65]
	C57BL/6 mice	20 mg/kg BW 5 times (every three days) via oral gavage before and 1 after irradiation	Amelioration of ROS production and premature senescence of hematopoietic stem cells	
Caffeic acid phenethyl esters	Sprague-Dawley rats	10 µmol/kg i.p. 30 min before irradiation	Prevention of oxidative and nitrosative damages induced by radiation	[69]
Chlorogenic acid	Human blood lymphocytes	0.5, 1, 2 and 4 μg/ml	Prevention of genomic instability induced by X-ray irradiation	[67]
	Mice	100, 200 and 400 mg/kg BW 1 or 24 h before irradiation	Increase of animal survival (with 200 mg/kg dose 1 h before irradiation with high doses of γ-radiation)	[68]
Curcumin	C57BL/6 mice	standard mouse chow (5015) with 1% or 5% curcumin *w*/*w*	Amelioration of radiation-induced pulmonary fibrosis Increase of mouse survival with no impairment of tumor cell killing by radiation.	[74]
	Wistar albino rats	100 mg/kg BW orally (by intra gastric intubation)	Protection against intestinal damage	[75]
	Wistar rats	150 mg/kg BW 1 day before irradiation to 3 days after orally	Prevention of radiotherapy-induced oxidative injury to the skin, through enhancing CAT, SOD, and GSH-Px	[78]
	Human blood cells	curcumin-encapsulated liposomes (up to 1046.5 µg/mL)	Reduction in the micronuclei frequency No genotoxicity	[80]
	Human foreskin fibroblast cells (HHF-2)	conjugated albumin based nanoparticles (50 µg/mL)	Increase in cell viability	[81]
	Balb/C mice	125, 250, 500 and 1000 mg/kg via tail vain	Increase in the survival rate	
Cyanidin-3-*O*-glucoside (C3G)	HaCaT keratinocytes	80, 160 and 200 μM	Suppression of COX-2 expression by interaction with the MAPK and Akt signalling pathways	[60]
	Kunming mice	125 µM, 250 µM and 500 µM chitosan-C3G nanoparticles; 500 µM C3G	Reduction of UVB-induced epidermal damage through the p53-mediated apoptosis signalling pathway Higher efficiency of nanoparticles respect to C3G at the same dose	[61]
	HaCaT keratinocytes	80, 160 and 200 μM	Intracellular decrease of ROS and of the phospho-p53 and phospho-ATM/ATR levels Expression of anti-apoptotic protein B-cell lymphoma 2	[59]
Epigallocatechin-3-*O*-gallate	C57 BL/6 J mice	12.5 or 25 mg/kg BW for 5 days	Reduction of IR-induced cell death in intestinal epithelial cells through Nrf2 Repression of IR-induced apoptosis and ferroptosis Amelioration of intestinal injury induced by total body irradiation	[49]
	Kunming mice	6.25, 12.5 and 25 mg/kg BW for 30 days	Prevention of the immune system damage Enhancement of the activity of enzymatic antioxidants (e.g., SOD, GSH-Px)	[50]
	Normal fetal lung fibroblasts (MRC5) and adult skin fibroblasts (84BR) Normal human epidermal keratinocytes (NHEK)	Up to 1 mM 250 μM	Prevention against ultraviolet radiation-induced DNA damage	[48]
Ferulic acid	Swiss mice	50, 75 and 100 mg/kg BW i.p. 1 h before irradiation	Faster repair of DNA	[66]
Flavopiridol *	A172/mp53 and HeLa/bcl-2 cells (radioresistant through genetic alteration)	0.0125–0.125 μM	Radio-sensitization via inhibition of sublethal damage and DNA double-strand breakage repair	[58]
Genistein	Human embryo liver cells (L-02)	1–20 μM	Low concentration: protection against radiation damage High concentration: radiosensitizing features	[52]
	Huh-7, Hep3B and Hep G2 human HCC cells L-02 cells	0–40 μM	Low dose (5 μM): HCC cell sensitivity enhancement to X rays; no significant toxicity to L-02 cells	[53]
	CD2F1 mice	100, 200, or 400 mg/kg BW s.c.	Protection against acute radiation injury (administered 24 h before irradiation) Hypothesized indirect mechanism (e.g., cytokine release)	[54]
	CD2F1 mice	150 mg/kg BW i.m.	Selective binding to estrogen receptor β	[55]
	Sprague-Dawley rats	5 mg/kg BW i.p. for 7 days	Up-regulation of ER-β and FOXL-2 Downregulation of TGF-β expression Reversion of radiotherapy-induced premature ovarian failure	[56]
Monoglucosyl rutin *	CHO 10B2 cells	0.001–0.1%	Increase in survival of IR-treated cells at doses greater than 2 Gy	[32]
Naringin	Swiss albino mice	75 mg/kg BW for 3 days	Reversion of the liver IR-induced redox-imbalance	[41]
Quercetin	CBA mice	100 mg/kg BW i.p. for 3 days	Protection of mice white blood cells from lethal effects and DNA damage before γ-irradiation	[35]
	Human white blood cells	50 µM	Protection from DNA damage after γ-irradiation	[34]
Quercetin glycosylated derivatives	Cell-free systems	10 µM	Reduction of DNA double-strand breaks	[33]
Rosmarinic acid	Mice	100, 200 and 400 mg/kg BW (oral) for 10 days (3 before and 7 after irradiation)	Promotion of the recovery of peripheral blood cells Enhancement of 30-day survival rates	[71]
Rutin + podophyllotoxin	C57BL/6 mice	2.5mg/kg BW 1 h before irradiation	ROS levels reduction Protection of cellular macromolecules Activation of antioxidant signaling pathway	[30]
	HaCat cells	0.025–0.4 µg/mL culture medium	Reduction of the cellular damage by scavenging free-radicals, cell-cycle arrest and DNA repair enhancement in the hematopoietic system	[29]
	Strain ‘A’ mice	2.5mg/kg BW i.m. 1 h before irradiation		
VND3207 vanillin derivative *	Human intestinal epithelial cells (HIEC)	40 µM	Promotion of intestinal repair after radiation injury by regulation of the DNA-PKcs pathway	[64]
	C57BL/6 J, NOD-SCID (Prkdcscid/scid) and BALB/c mice	100 mg/kg BW i.g. 30 min before irradiation		
	C57BL/6J mice	100 mg/kg BW (oral gavage) 30 min before/after irradiation	Intestinal repair after radiation injury by reduction of ROS-induced DNA damage and modulation of activated p53 levels in intestinal epithelial cells	[63]

* semi-synthetic compound.

## Abbreviations

ACE	Angiotensin Converting Enzyme
Akt	Protein kinase B
Bax	Bcl-2-associated X protein
Bcl-2	B-cell lymphoma 2
CAT	Catalase
CDK	Cyclin-Dependent Kinase
COX-2	Cyclo-oxygenase-2
DDR	DNA Damage Response
DPPH	2,2-diphenyl-1-picryl hydrazyl
DSB	Double Strand Break
e−_aq_	Hydrated electrons
EGCG	Epigallocatechin-3-*O*-gallate
ERK	Extracellular signal-regulated kinase
ER-β	Estrogen Receptor-β
FOXL-2	Forkhead box L2 protein
G-CSF	Granulocyte-Colony Stimulating Factor
GM-CSF	Granulocyte Macrophage-Colony Stimulating Factor
GPx	Glutathione peroxidase
GRP78	78-kDa Glucose-Regulated Protein
GSH	Reduced Glutathione
HO-1	Heme Oxygenase-1
HO_2_^●^	Hydroperoxyl radical
HSCs	Hematopoiesis Stem Cells
IL-1	Interleukin-1
IR	Ionizing Radiation
LPS	Lipopolysaccharide
MAPK	Mitogen-Activated Protein Kinase
MKP3	MAP Kinase Phosphatase 3
NF-κB	Nuclear factor kappa B
Nrf2-ARE	NF-E2-related factor 2-Antioxidant Responsive Element
O_2_^−●^	Superoxide anion radical
PSHE	*Pterocarpus santalinus* hydroalcoholic extract
PUMA	p53 Up-regulated Modulator of Apoptosis
RNF8	Ring Finger Protein 8
ROS	Reactive Oxygen Species
SIRT1	Sirtuin 1
SOD	Superoxide Dismutase
SSB	Single Strand Break
TGF-β	Transforming Growth Factor-β
TNF-α	Tumor Necrosis Factor-1
UHPLC-HRMS/MS	Ultra High Performance Liquid Chromatography-High Resolution Tandem Mass Spectrometry
